# Parental Attachment and Psychosocial Adjustment in Adolescents Exposed to Marital Conflict

**DOI:** 10.3390/children11030291

**Published:** 2024-02-29

**Authors:** Jesús Maya, Isabel Fuentes, Ana Isabel Arcos-Romero, Lucía Jiménez

**Affiliations:** 1 Department of Psychology, Faculty of Psychology and Education, Universidad Loyola Andalucía, 41704 Seville, Spain; jmmaya@uloyola.es (J.M.);; 2 Department of Developmental and Educational Psychology, University of Seville, 41018 Seville, Spain

**Keywords:** parental attachment, adolescents, marital conflict, behaviour problems, emotional intelligence

## Abstract

(1) Background: Interparental conflict is a phenomenon that poses a serious threat not only to the quality of life of the couple but also to the father–child relationship, mother–child relationship, and well-being of adolescents. This study examined the difference in parental attachment and adjustment in adolescents exposed to marital conflict versus those not exposed to parental conflicts in low-income areas. (2) Methods: 67 adolescents involved in Child Welfare Services (CWS) in disadvantaged neighbourhoods in southern Spain were examined. The sample was split into two groups according to the exposure to marital conflict. Subsequently, differences between groups were analysed on father attachment, mother attachment, aggression, antisocial behaviour, and emotional intelligence. (3) Results: Primarily, the data showed significantly worse attachment with the father among conflict-exposed versus non-conflict-exposed adolescents. These results were not found for the attachment with the mother. Additionally, greater anger and worse stress management were found in conflict-exposed adolescents. (4) Conclusions: The results partially confirmed the spillover and compensatory hypothesis. Practical implications point out that developing preventive interventions that protect the father–adolescent attachment in situations of family conflict is recommended.

## 1. Introduction

Family systems theory proposes that the family system is composed of different interrelated subsystems: marital, parental, and fraternal [1]. These subsystems cannot be understood as independent entities, but to understand the family context and the development of their individuals, the relationships, and influences between subsystems must be addressed [1]. Specifically, the marital or inter-parental subsystem refers to the relationships among the caregivers of the child. This subsystem exists prior to the birth of the child and is maintained during childhood and adolescence. In comparison to the parental subsystem, the inter-parental subsystem is expected to be characterized by symmetry in the relationship. Thus, couple relationships should present a functioning negotiated by the members of the couple [2]. These norms determine the way in which these individuals perceive themselves and each other. When a behaviour differs from the established interaction pattern model, it can generate tension and confrontation in the couple. The parent–child relationship is not independent of the existing tensions in the couple relationship. The influence between the parental subsystem and the marital subsystem is bidirectional [3]. In this way, conflicts in the couple can have consequences in the parental relationship and in the adjustment of adolescents who grow up in these families. In fact, identifying the dimensions affected by marital conflict can be of great interest to develop interventions and treatments at the family and individual levels and ultimately to protect children and adolescents from marital conflicts.

Marital conflict refers to a mutual opposition between the members of the couple that reflects differences among them [4]. Given these differences, some couples are able to generate effective coping mechanisms to restore balance and well-being in the system. However, others present greater difficulties in seeking solutions, which causes even more problematic events within the marital subsystem and the family system [5]. 

Conflict is unavoidable, and some authors suggest that it can even have a constructive function through cooperative interactions [2]. Constructive conflicts are characterized by a warm environment in which both parties focus on the search for common agreements, express their feelings, provide positive feedback, respect different points of view and could reach the most cost-effective and constructive solution [6]. Likewise, constructive processes involve cooperative interaction patterns and effective problem-solving behaviours between partners in which both understand the needs and demands of the other [7].

However, conflict can also be interpreted as a source of stress between partners when it negatively interferes with their quality of life [8]. Destructive conflict is used when a disagreement between partners is characterized by the appearance of negative emotions and behaviours such as anger, verbal, and nonverbal hostility, and/or physical aggression. The main indicators refer to arguments, insults, threats, contempt, shouting and/or abuse between parents [9]. These conflicts lie in ineffective resolution behaviours, negative cognitive evaluations, incongruent expectations, and poorly adaptive coping strategies [7]. The reasons for this conflict can be very diverse: the economy, housework, family relationships, differences in values, expectations about their relationship, sexuality, or education of children, among others [7].

Different theories explain the influence of marital conflict on parent–child relationships and on adolescent mental health. At the behavioural level, following the social learning theory [10], children exposed to marital conflict can learn to perpetrate a series of aggressive behaviours in their interpersonal relationships with peers. According to the cognitive-contextual model [11,12], the exposure of children to inter-parental conflict can favour the appearance of a series of negative attributes, such as a greater perception of threat and self-blame feelings. According to the theory of emotional security [6,13], children need to perceive that their entire family system is safe, in addition to establishing a positive relationship with their attachment figures.

Marital conflict has several implications at both the family and parental levels. There is a significant relationship between the parental role and that of a partner since a positive marital relationship is associated with better parental competence [14]. However, when there is a confrontation between the members of the couple, the attention and supervision of the child can be neglected [15]. In fact, the spillover hypothesis argues that anger and hostility associated with inter-parental conflict can make parents less patient and more reactive towards their children [8]. It is observed that adolescents exposed to episodes of marital conflict value the relationship they have with their parents less positively and seek less affection and emotional support from them [16]. Likewise, conflicts between parents can make adolescents feel guilty and divided, which in some cases causes them to seek security by establishing a preferential relationship with one of the two parents, which can cause a loyalty conflict [17]. From this perspective, the compensatory hypothesis indicates that parents exposed to conflicts in their relationship seek to spend more quality time with their children [18,19]. Kouros et al. [20] show data that support this compensatory hypothesis among mothers but not fathers.

In general, inter-parental conflict is related to a worse parent–child relationship and a worse bond between the two [6]. When considering parent gender (mother or father), low quality in the relationship (less affection and more conflict) is found especially with the fathers of children and adolescents exposed to marital conflict in comparison with the mothers of children and adolescents exposed to conflict [21,22]. However, other studies show poor quality of the parent–child relationship regardless of sex and a negative relationship with the father only after a divorce [23,24,25,26]. 

Regarding the psychosocial adjustment of children and adolescents exposed to conflict, it is known that marital conflicts can alter the emotional security of the child, which is manifested in emotional reactions of fear, anguish, and threat [27]. When young people perceive a possible risk to their emotional security, they show concern and react immediately to this situation or react with indifference [28]. Thus, those conflicts characterized by attitudes of aggression and anger between the parents are a serious risk factor for the development of maladaptive skills in children and adolescents [29]. When they are exposed to marital conflict, information processing before a certain episode with peers presents a certain tendency to hostility and anger [8]. Thus, the probability that those children exposed to conflict become in adolescents presenting aggressive behaviours and experiencing dysfunctional relationships is higher in comparison to other children [5]. However, according to the stress relief hypothesis [30], children exposed to high levels of marital conflict improve when divorce occurs and their exposure to conflicts decreases [31]. Moreover, the marital conflict will make it more difficult for the adolescent to establish a healthy self-concept, exhibiting difficulties in the development of his self-concept and self-care. Finally, the increase in problems during the adolescence period is related to inter-parental conflict and lower marital satisfaction [32,33]. 

Research has conceptualized the influence of marital conflicts on the family system and on the adjustment of children and adolescents. However, most of these studies have been generic and described parental relationships without addressing the specific father–adolescent and mother–adolescent bond. Additionally, most of the literature focused on analysing the influence of marital conflict on children reports data in middle-class contexts. Following the recommendations of Yu et al. [22], it is necessary to study marital conflict and its association with the father–adolescent and mother–adolescent relationship among low-income families. Additionally, according to Arenas-Rojas [34], families at psychosocial risk are in a situation of vulnerability as a result of the presence of multiple risk factors, such as the characteristics of parents and children, the patterns of interaction and relationships, and the characteristics of the neighbourhood and the community. In relation to parental figures, the presence of a low educational level and/or a precarious work situation, the diagnosis of a psychological disorder and/or problems related to consumption can be considered risk factors. In relation to the community context, risk factors include a negative economic situation, a high dependence on social assistance, high levels of community violence, and a precarious place of residence that endangers the integrity of its members and their relationships [34].

In situations of psychosocial risk, it is possible that the quality of the couple relationship is influenced by stressors. When the members of the couple face a series of stressful events, these events negatively affect the quality of that relationship, increasing the frequency of conflicts and arguments [34]. Therefore, it is not strange to observe how in families at psychosocial risk, satisfaction with the couple relationship is at lower levels with respect to the average [34]. Similarly, there is a high degree of marital instability and multiple separations between the members of the couple [35]. In a complementary way, a good relationship within a context of psychosocial risk can act as a moderator of the influence exerted by stressful life events in the family system. Thus, the quality of the relationship can influence how a situation of psychosocial risk affects the development of adolescent children [34].

Given the scarcity of studies on the impact of marital conflict within parent-adolescent and mother–adolescent dyads, particularly in psychosocially at-risk families, the research question is: Are there differences in parental attachment and emotional and behavioural adjustment between adolescents exposed to marital conflicts and those not exposed in disadvantaged areas? This study has two specific objectives: (a) to compare parental attachment in the mother–adolescent and father–adolescent dyads between adolescents exposed to marital conflict and unexposed adolescents and (b) to analyse adolescents’ psychosocial adjustment by comparing aggressiveness, antisocial behaviour, and emotional intelligence among adolescents exposed to marital conflict and unexposed adolescents. As the main hypothesis, according to the available evidence, it is proposed that adolescents exposed to marital conflicts in risk areas will show worse attachment with both their mother and their father as well as worse psychosocial adjustment compared to those adolescents not exposed to this type of conflict.

## 2. Materials and Methods

### 2.1. Participants

The sample consisted of 67 adolescents (46.3% boys) aged from 11 to 18 (*M* = 14.37; *SD* = 1.51) involved in Child Welfare Services (CWS) and living in low-socioeconomic-status neighbourhoods in southern Spain. The following inclusion criteria were considered: (a) belonging to families living in disadvantaged neighbourhoods; (b) being enrolled in CWS due to the existence of family conflict and difficulties in parenting, (c) exhibiting problematic behaviour in school (absenteeism, expulsions from school, or conflicts with peers); and (d) being recruited as eligible for a family therapy treatment aimed to improve family relationships and reduce behavioural problems [36]. A total of 89.6% of the adolescents were Spanish, and the others were from Colombia, Algeria, Peru, Romania, and Russia. Thirty-one adolescents (46.3%) were witnesses of conflict between their parents (defined as fights, shouting, and strong and recurring fights between their parents) compared to 36 adolescents (53.7%) who did not report marital conflict in the family environment. Regarding the comparison between groups, there were no significant differences (χ^2^ = 1.802; *p* > 0.05) in terms of gender: 42.4% boys and 57.6% girls in adolescents exposed to marital conflicts and 58.8% boys and 41.2% girls in adolescents not exposed to marital conflicts. Furthermore, there were no differences in terms of age (*t* = 0.282; *p* > 0.05): mean age was 14.15 in adolescents exposed to conflicts and 14.26 in adolescents not exposed to marital conflicts.

### 2.2. Instruments

Sociodemographic information. An ad hoc questionnaire was designed to report the sociodemographic profile of the adolescents, including sex, age, educational level, and nationality.

Attachment with the mother and the father. Inventory Parents-Peer Attachment (IPPA) [37,38]. This instrument has two versions: attachment with the mother and attachment with the father. Both versions are composed of 25 items, identical but changing the word mother for father depending on the version. The response scale ranged from 1 = never or almost never to 5 = almost always or always. The inventory analyses attachment through three dimensions: communication (e.g., “My father senses when I’m upset about something”), trust (e.g., “My father is a good father”), and alienation (e.g., “I feel angry with my father”). In the communication and trust dimensions, higher scores are interpreted as a secure attachment. However, in the alienation dimension, lower scores are interpreted as indicating a better attachment. The sum of the scores for the communication and trust dimensions, minus the alienation score, provides the total parental attachment score. Higher scores are interpreted as indicating stronger parental attachment. Confirmatory analysis of the IPPA inventory in Spanish adolescents found that three factors (communication, trust, and alienation) showed a good fit for both the mother (χ^2^/df = 2.123, RMSEA = 0.062, CFI = 0.907, TLI = 0.895) and the father version (χ^2^/df = 2.436, RMSEA = 0.078, CFI = 0.903, TLI = 0.892) [38]. The internal reliability for the version of attachment with the mother was communication (α = 0.77), trust (α = 0.88), alienation (α = 0.62), and total attachment (α = 0.80). For the version of attachment with the father, reliability was as follows: communication (α = 0.87), trust (α = 0.95), alienation (α = 0.55), and total attachment (α = 0.89).

Aggression. The Aggression Questionnaire (AQ) [39]. This instrument includes 29 items measured on a Likert-type scale, where 1 = extremely uncharacteristic of me and 5 = extremely characteristic of me. It consists of four subscales: physical aggression (e.g., “Once in a while, I can’t control the urge to strike another person”), verbal aggressiveness (e.g., “My friends say that I’m somewhat argumentative”), anger (e.g., “I flare up quickly but get over it quickly”), and hostility (e.g., “I sometimes feel that people are laughing at me behind my back”). A higher score implies greater aggressiveness. A confirmatory analysis in Spanish adolescents showed that the four-factor model provides a good fit (GFI = 0.928; AGFI = 0.916; RMSEA = 0.047) [39]. The reliability of the subscales was as follows: physical aggressiveness (α = 0.82), verbal aggressiveness (α = 0.64), anger (α = 0.61), and hostility (α = 0.65).

Antisocial behaviour. The Questionnaire of Antisocial-Criminal Behaviours (AD) [40]. In this study, we used only the antisocial behaviour scale, whose items showed adequate loadings on the scale in its original validation [40]. This scale consists of 20 items with two response options (yes/no), which collect different behaviours that the adolescent has performed in the past two months (e.g., “breaking or throwing things that belong to other people”). Higher scores imply greater antisocial behaviour.

Emotional Intelligence. The Emotional Quotient—Youth Version (EQ-YV) [41]. An adaptation of the translation recommended by the original authors was carried out by the research team. This questionnaire consists of 60 items measured on a Likert-type scale from 1 = Very seldom true or not true of me to 4 = Very often true of me or true of me. It evaluates the dimension of emotional intelligence through five subscales: intrapersonal intelligence (e.g., “I can easily talk about my feelings”), interpersonal intelligence (e.g., “I am able to respect others”), adaptability (e.g., “I can understand difficult questions”), stress management (e.g., “I can be calm when I am angry”), and mood (e.g., “I feel good about myself”). Higher scores in each dimension are interpreted as a positive indicator. This instrument has demonstrated its internal validity and the good fit of its four scales (intrapersonal, interpersonal, adaptability, and stress management) and the mood scale across various samples of adolescents [42,43]. The internal consistency of the dimensions was the following: intrapersonal intelligence (α = 0.63), interpersonal intelligence (α = 0.76), adaptability (α = 0.78), stress management (α = 0.79), and mood (α = 0.89).

### 2.3. Procedure

A cross-sectional quantitative design was followed to compare two groups of adolescents as a function of exposure to episodes of interparental conflict. This research was developed in accordance with the formal consent of the regional committee (Code 0985-M1-18) and followed the standards of the Declaration of Helsinki. The data for this research were obtained from adolescents who participated in Scene-Based Psychodramatic Family Therapy [36]. This is a group therapy consisting of 10 sessions that have been implemented in underprivileged neighbourhoods in southern Spain since 2019. It is aimed at adolescents exhibiting problematic behaviours in school and family settings who are under the care of CWS, with the objective of enhancing family relationships and reducing conflicts in behaviours. A total of 86 adolescents took part in the therapy, but only 67 were selected because the remaining adolescents either did not complete all the questionnaires or did not attend session 2, where the questionnaires were distributed. Participation in the evaluation was voluntary with the informed consent of their parents. A researcher from the team collected the data and explained the study and clarified any doubts. Likewise, the anonymous and confidential nature of the investigation was explained and guaranteed. The adolescents completed the questionnaires individually for approximately 30 min. The questionnaires were self-administered in the following order: sociodemographic questionnaire, attachment questionnaire (mother version and father version), antisocial behaviour questionnaire, aggressiveness questionnaire, and emotional intelligence questionnaire.

### 2.4. Data Analysis

The assumptions of normality, asymmetry, and kurtosis were tested for each variable. These analyses were performed independently for each group (i.e., adolescents exposed to conflict and unexposed adolescents), and the results were satisfactory. The comparison analyses between groups across gender and age were carried out by using crosstabs. Then, differences in parental attachment, aggressiveness, antisocial behaviour, and emotional intelligence were explored between the two groups using Student’s *t*-test for independent samples. The significance level of *p* < 0.05 was examined and the effect size was considered by observing Cohen’s d value. Following Cohen [44], we considered the magnitude of the effect as small when values are <0.30, medium when values are between 0.50 and 0.80, and large when these values are greater than >0.80, approximately.

## 3. Results

### 3.1. Mother and Father Attachment of Adolescents Exposed to Marital Conflict

Concerning the attachment with the mother, non-statistically significant differences were observed between adolescents exposed to marital conflict and those not exposed in the overall points nor in the dimensions of trust and family communication with the mother (see Table 1). Significant differences were found only in the alienation dimension, with adolescents exposed to interparental conflicts exhibiting a higher level of alienation in comparison to adolescents not exposed to conflict (*t* = −2.05; *p* = 0.044). The magnitude of the effect was medium. 

Regarding father attachment, the results showed statistically significant differences between adolescents exposed to marital conflict and those not exposed in the total score and in all attachment dimensions. Those adolescents exposed to paternal conflict reported lower levels of global attachment with the father (*t* = 5.20; *p* = 0.000), parental communication (*t* = 4.93; *p* = 0.000), and trust (*t* = 3.99; *p* = 0.000) and higher levels of alienation (*t* = −4.44; *p* = 0.000) than adolescents not exposed to marital conflict between their parents (see Table 2). In all the cases, the magnitude of the effect was large.

### 3.2. Psychosocial Adjustment of Adolescents Exposed to Marital Conflict

Statistically significant differences were found between adolescents exposed to marital conflict and those not exposed to aggressiveness; with adolescents exposed to episodes of marital conflict presenting significantly higher levels of anger than unexposed adolescents (*t* = −2.88; *p* = 0.005). The magnitude of the effect was very large. No statistically significant differences were found between both groups in antisocial behaviour (see Table 3). In relation to emotional intelligence, statistically significant differences were found only in the stress management subscale; with adolescents exposed to interparental conflict presenting greater difficulties in stress management than those not exposed (*t* = 2.32; *p* = 0.024). In this case, the magnitude of the effect was medium.

## 4. Discussion

The general objective of this study was to compare mother–adolescent and father–adolescent attachment as well as psychosocial adjustment between adolescents exposed to marital conflicts and adolescents not exposed to this type of conflict. This objective was specifically explored in a sociocultural setting and a sample with little prior evidence as Spanish adolescents from CWS living in disadvantaged neighbourhoods. Families at psychosocial risk are usually characterized by a high rate of interparental conflict [45], an aspect that coincides with what was found in this study, where almost half of the adolescents were exposed to interparental conflicts. Considering that the quality of the marital relationship is an important predictor of the quality of family dynamics and adolescent development, particularly in risky areas [34], these results support the importance of developing preventive measures to improve the quality of family relationships.

Regarding the mother–adolescent and father–adolescent dyadic relationships, the data revealed that the impact of marital conflict on parental attachment varies depending on the parental figure. Our data suggested that interparental conflict affects the relationship established by the adolescent with the father figure to a greater extent. There has been controversy in the literature about the differential impact of marital conflict on the relationship of the adolescent with his mother and father [22,23,24,25,26]. More evidence is available indicating that marital conflict equally affects the relationship with the mother and the father [23,25,26]. However, our results support those studies reporting that marital conflict affects the relationship with the father to a greater extent [21,22]. There is some evidence indicating that in a situation of family stress, adolescents need to find security in one of their parental figures [17]. Considering the risk situations in which the research was developed, it is possible that these adolescents had a greater need for a secure attachment to face the family and community stress they are exposed to. Likewise, in contexts of risk where job insecurity is high, it is possible that the adolescent perceives the vulnerability of the mother and shows greater concern for her well-being [17]. In this regard, Iraurgi et al. [12] state that in situations of marital conflict, children seem to adopt triangulation strategies to cope with the discomfort that is caused. From the systemic theory, Minuchin [1] already expressed that in situations of conflicting loyalties, children usually adjust to a process of triangulation that can lead to other problems. This phenomenon indicates that adolescents experience division among their parents which means aligning with one parent against the other [17,46]. Attaching to the mother, as occurs in situations of separation after exposure to marital conflict [23,24], can result in paradoxical well-being for the adolescent who obtains short-term support, but in the medium-long term, this can have consequences in his adjustment given the distance from the father and the loss of a quality relationship with one of the two parental figures. Following the systemic theory [1], the adolescent exposed to a coalition with the mother against the father can adopt a role of parentification or spousal role with respect to his mother, which has been associated with adjustment problems. However, the fact that the adolescent shows an improved relationship with his mother may constitute partial support for the compensatory hypothesis [18,19], which establishes that, in situations of couple conflict, one of the members of the couple will seek to spend more quality time with the children. Thus, in risky contexts characterized by job insecurity, it is possible that women can spend more time at home in favour of greater attachment to their children. 

Continuing with the psychosocial adjustment of adolescents exposed to conflict, the spillover hypothesis [8] proposes that situations of tension between the couple can be transmitted to the children and affect their behaviour. According to this hypothesis, there are greater differences between adolescents exposed to conflicts and nonexposed adolescents in the dimension of anger and stress management.

In relation to anger and stress management, our results are partially consistent with the evidence about how exposure to situations of marital conflict is related to a greater predisposition in adolescents to aggression and hostility [47,48]. According to modelling theory [10], adolescents exposed to interparental conflict have probably learned that aggressive behaviour is an appropriate way to solve problems and, therefore, tend to behave in the same way in their interactions [4]. Thus, the exposure of adolescents to episodes of conflict between their parents contributes to the processing of information before a certain event with their peers which presents a certain tendency to hostility and anger. However, this relationship is influenced by the way in which the conflict is expressed. It is possible in those families in which frequent but less intense conflict between parents does not have as negative an influence on the children compared to conflict between parents that includes behaviours such as physical aggression [4,49]. Our data did not show differences between groups in dimensions such as intrapersonal intelligence, interpersonal intelligence, or antisocial behaviour, but did on anger and stress management. Dimensions such as emotional intelligence can be more resistant to change, with stress management being a specific complication. In scenarios of exposure to conflict, might be a deficit of resources for stress management. These results are consistent with the evidence that supports the direct influence that inter-parental conflict has on the increase in the stress levels of children and adolescents [50,51,52]. Moreover, although exposure to the conflict may increase the levels of anger among the exposed group compared to the unexposed group, this increase in anger may not be accompanied by a greater manifestation of antisocial behaviour. Emotional intelligence can act as a buffer for this result. Also, the finding that attachment with the mother presents similar levels in both groups may indicate that this relationship is a protective factor for the exhibition of antisocial behaviours. In fact, previous studies have found that positive attachment with the mother acts as a protective factor in antisocial behaviours [53].

This study has several limitations. First, the results and data generalizability should be taken with caution due to the limited number of participants. The study has a medium sample size given the specificity of the sample, which makes it difficult to compare other variables, such as father–son, father–daughter, mother–son, and mother–daughter dyads. In addition, a dichotomous view of conflict is presented, with the analysis of the frequency and intensity of marital conflict left for future research. Likewise, it is unknown whether the marital conflict is symmetrical or asymmetrical, as well as the power differences among the individuals in the couple. A multi-informant evaluation design that includes the point of view of the fathers and mothers could shed some light. 

However, despite these limitations, the study is innovative and has strengths such as the focus on adolescents being attended by CWS, the conduct of research in low-income neighbourhoods, and the analysis of emotional, behavioural, and relational dimensions of adolescents, with an emphasis on the differentiated bond between father and mother.

## 5. Conclusions

The results of this study show the importance of marital conflict, particularly for father attachment, anger, and stress management among adolescents enrolled in CWS who live in disadvantaged neighbourhoods. The dyadic analysis of mother–adolescent and father–adolescent attachment is highlighted, as well as the challenge of doing research in psychosocial risk contexts. The attachment with the father is the highest significant dimension when comparing adolescents exposed to conflicts versus nonexposed adolescents. In this sense, worse communication, trust, and attachment with the father have been observed in adolescents exposed to conflicts compared to those not exposed. These data are not replicated when considering the attachment to the mother. This result implies the need to involve fathers, particularly in risky contexts, in both preventive parenting programs and psychotherapeutic processes. Additionally, according to Devonshire et al. [54], couple and family interventions aimed at constructive conflict resolution are recommended. At the psychotherapeutic level, in line with Minuchin [55], a systemic approach to address family difficulties is supported. It is essential to differentiate the parental role and the conjugal role and to protect parent–adolescent attachment in conflict situations. Likewise, the higher levels of anger and the difficulty in managing stress among adolescents exposed to marital conflict are consistent with the spillover hypothesis of the transmission of tensions from partners to children and, therefore, are an indicator of the need to develop actions with adolescents that can act as protective factors against family stress.

## Figures and Tables

**Table 1:** Differences in the mean scores of maternal attachment between adolescents exposed to marital conflict and unexposed adolescents.

	Marital Conflict	*M*	*SD*	*t*	*p*	*d*
Communication	Yes	30.26	8.04	0.63	0.531	
	No	31.56	8.04			
Trust	Yes	33.77	9.83	1.60	0.115	
	No	37.47	9.09			
Alienation	Yes	17.48	4.25	−2.05	0.044 *	0.51
	No	14.97	5.57			
Total	Yes	46.55	19.13	1.60	0.114	
	No	54.06	19.10			

Note. *M* = mean; *SD* = Standard deviation; *t* = Student’s t; *d* = Cohen’s d. * *p* < 0.05.

**Table 2:** Differences in the mean scores of paternal attachment between adolescents exposed to marital conflict and unexposed adolescents.

	Marital Conflict	*M*	*SD*	*t*	*p*	*d*
Communication	Yes	20.18	8.63	4.93	0.000 ***	−1.41
	No	32.11	8.26			
Trust	Yes	25.45	11.42	3.99	0.000 ***	−1.14
	No	37.67	10.00			
Alienation	Yes	18.36	4.48	−4.44	0.000 ***	1.26
	No	13.00	3.98			
Total	Yes	27.27	20.57	5.20	0.000 ***	−1.48
	No	56.78	19.16			

Note. *M* = mean; *SD* = Standard deviation; *t* = Student’s t; *d* = Cohen’s d. *** *p* < 0.001.

**Table 3:** Differences in the mean scores of aggressive behaviour, antisocial behaviour, and emotional intelligence between adolescents exposed to marital conflict and nonexposed adolescents.

	Marital Conflict	*M*	*SD*	*t*	*p*	*d*
Aggression						
Physical aggression	Yes	29.93	8.18	−1.80	0.076	
	No	25.83	9.61			
Verbal aggressiveness	Yes	14.50	4.44	−1.79	0.078	
	No	12.56	4.21			
Hostility	Yes	23.11	4.39	−1.81	0.075	
	No	20.47	6.64			
Anger	Yes	24.93	0.72	−2.88	0.005 **	5.13
	No	20.97	0.82			
Antisocial behaviour						
Antisocial behaviour	Yes	9.93	4.37	−1.83	0.072	
	No	7.66	5.26			
Emotional intelligence						
Intrapersonal intelligence	Yes	14.25	3.85	0.90	0.370	
	No	15.14	3.94			
Interpersonal intelligence	Yes	38.25	5.32	0.56	0.576	
	No	39.11	6.59			
Adaptability	Yes	30.00	4.09	0.22	0.826	
	No	30.29	5.77			
Stress management	Yes	24.75	5.96	2.32	0.024 *	−0.60
	No	28.74	7.39			
Mood	Yes	43.64	7.77	1.05	0.298	
	No	45.80	8.34			

Note. *M* = mean; *SD* = Standard deviation; *t* = Student’s t; *d* = Cohen’s d. * *p* < 0.05; ** *p* < 0.01.

## Data Availability

The data presented in this study are available on request from the corresponding author. The data are not publicly available due to the sample included minors.
